# The potential of repurposing clemastine to promote remyelination

**DOI:** 10.3389/fncel.2025.1582902

**Published:** 2025-05-07

**Authors:** Reiji Yamazaki, Nobuhiko Ohno

**Affiliations:** ^1^Department of Anatomy, Division of Histology and Cell Biology, School of Medicine, Jichi Medical University, Shimotsuke, Japan; ^2^Division of Ultrastructural Research, National Institute for Physiological Sciences, Okazaki, Japan

**Keywords:** remyelination, oligodendrocyte, clemastine, white matter injury, drug repositioning

## Abstract

White matter in the central nervous system comprises bundled nerve fibers myelinated by oligodendrocytes. White matter injury, characterized by the loss of oligodendrocytes and myelin, is common after ischemic brain injury, inflammatory demyelinating diseases including multiple sclerosis, and traumatic damage such as spinal cord injury. Currently, no therapies have been confirmed to promote remyelination in these diseases. Over the past decade, various reports have suggested that the anti-muscarinic drug clemastine can stimulate remyelination by oligodendrocytes. Consequently, the repurposing of clemastine as a potential treatment for a variety of neurological disorders has gained significant attention. The therapeutic effects of clemastine have been demonstrated in various animal models, and its mechanisms of action in various neurological disorders are currently being investigated. In this review, we summarize reports relating to clemastine administration for white matter injury and neurological disease and discuss the therapeutic potential of remyelination promotion.

## 1 Introduction

Oligodendrocytes are a form of glial cell in the central nervous system (CNS) (Bercury and Macklin, 2015), which extend processes around nerve cell axons and generate myelin to enhance conduction velocity by saltatory conduction (Nave and Werner, 2014; Osanai et al., 2022). To ensure the rapid transmission of information required to maintain brain function, white matter tracts of the CNS consist predominantly of myelinated fibers (Fields, 2010), critical for facilitating communication between different brain regions (Filley and Fields, 2016; Ribeiro et al., 2024). White matter injury is brain damage characterized by the loss or demyelination of these fibers (Ohno and Ikenaka, 2019). Reduced blood flow to brain tissue also often results in white matter damage and the loss of oligodendrocytes (Wang et al., 2016; Youssef et al., 2021). Ischemic stroke is therefore a common cause, alongside demyelinating diseases such as multiple sclerosis (MS) (Compston and Coles, 2008; Wang et al., 2016; Reich et al., 2018). To study white matter injury, focal lesions have been generated in animal models, targeting tracts of the corpus callosum, internal capsule, and spinal cord (Blakemore and Franklin, 2008; Huang et al., 2011; Keough et al., 2015; Yamazaki et al., 2021). Therapies for diseases associated with white matter injury have focused on the induction of remyelination by oligodendrocytes (Ohtomo and Arai, 2020; Youssef et al., 2021; Huang et al., 2023). Pharmacological options that promote remyelination are important for functional recovery after white matter injury.

High-throughput screening platforms using micropillar arrays have been used to identify candidate medications for demyelinating diseases that promote remyelination by oligodendrocytes; clemastine was first identified as a potential remyelinating agent in this way by Mei et al. (2014). Clemastine and its salt form, clemastine fumarate, are first-generation antihistamines used to treat allergy symptoms and relieve itching, with sedative and anticholinergic effects (Simons, 2004). Clemastine fumarate has enhanced solubility and bioavailability, and so is the commonly used form for pharmaceutical purposes, basic research, and clinical settings. Because it is already widely used in the clinic, the potential repurposing of the drug for the treatment of white matter injury and other neurological diseases has recently gained attention (Jiang et al., 2023; Zhu et al., 2023).

In this review, we summarize the therapeutic potential of clemastine in white matter injury and neurological diseases, including the latest findings and insights.

## 2 Therapeutic effects of clemastine in animal models and potential molecular mechanisms

**Figure 1:**
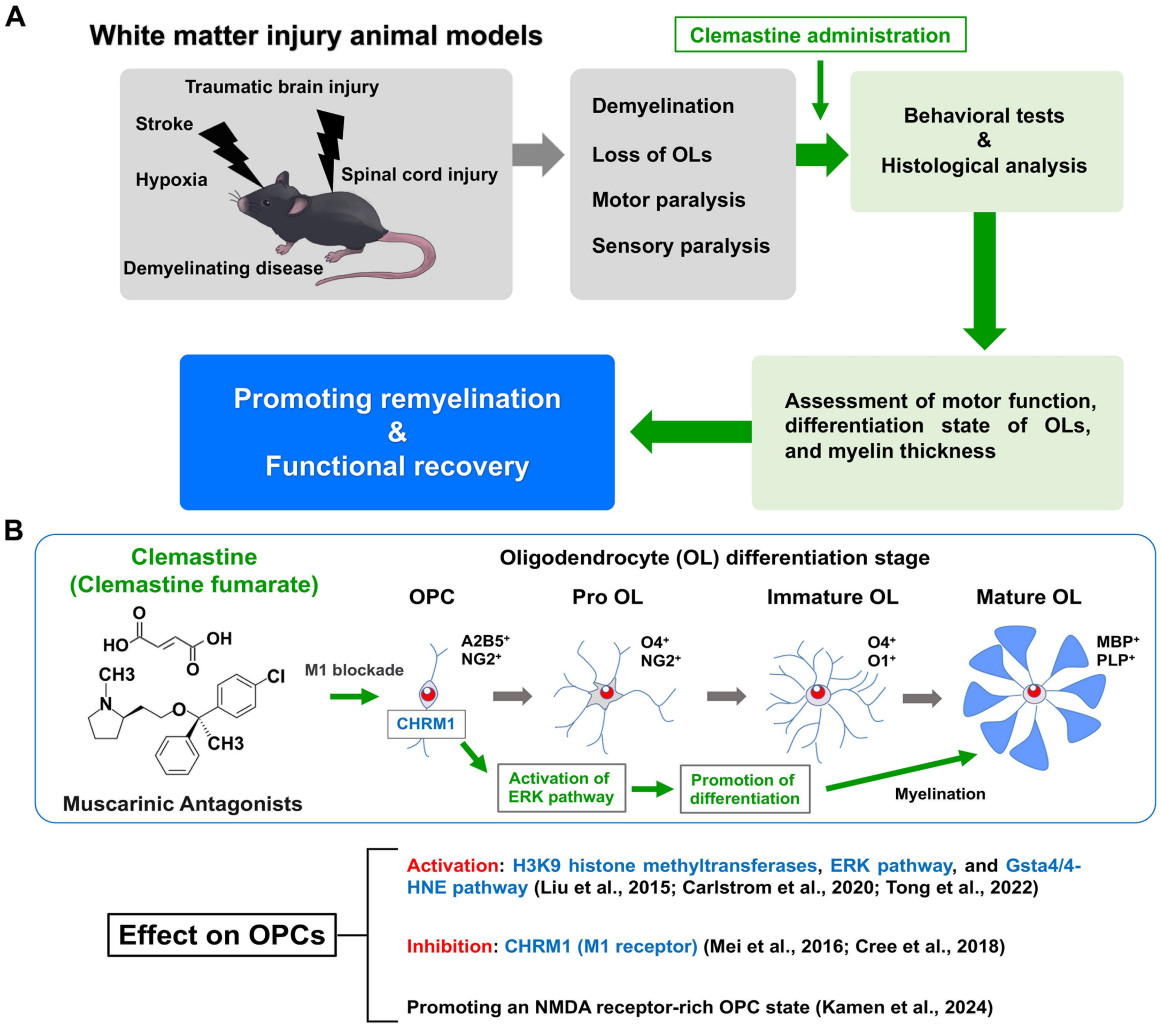
Therapeutic effects of clemastine in a mouse model of white matter injury and molecular mechanisms of action in oligodendrocyte precursor cells. **(A)** The animal models of white matter injury are induced demyelination, oligodendrocyte loss, motor paralysis and sensory paralysis. Motor function and remyelination state of oligodendrocytes (OLs) are assessed by behavioral tests and histological analysis. Clemastine administration in these animal models promotes remyelination and functional recovery. **(B)** Clemastine administration activates the extracellular signal-regulated kinase (ERK) pathway to promote differentiation via inhibition of the M1 muscarinic acetylcholine receptor (CHRM1) in oligodendrocyte precursor cells (OPCs). Clemastine also induces the activation of H3K9 histone methyltransferases and the glutathione S-transferase 4α (Gsta4)/4-hydroxynonenal (4-HNE) pathway in OPCs. In addition, clemastine promotes the *N*-methyl-d-aspartate (NMDA) receptor-rich state in OPCs. Shown are the schematic diagram of oligodendrocyte (OL) differentiation and stage-specific markers.

Since being identified for potential repurposing, clemastine has been administered in various animal models associated with demyelination (Figure 1A).

### 2.1 Summary of the effects of clemastine on remyelination in different animal models

Lysophosphatidylcholine (LPC)-induced demyelination is a representative animal model in which demyelination lesions are generated by focal injection; the model is highly informative for the assessment of remyelination (Blakemore and Franklin, 2008; Keough et al., 2015). Clemastine was shown to promote remyelination in an LPC-induced mouse model of spinal cord demyelination (Mei et al., 2014; Jensen et al., 2018). Recently, we have developed a mouse model of internal capsule demyelination that allows the evaluation of remyelination-induced functional recovery (Yamazaki et al., 2021; Yamazaki and Ohno, 2024). In this model, an asymmetric motor deficit is induced by internal capsule demyelination; recovery is associated with subsequent remyelination (Yamazaki et al., 2021; Yamazaki and Ohno, 2024). Using this model, we have also shown that clemastine administration promotes remyelination and related functional recovery (Yamazaki et al., 2023). A mouse model of demyelination induced by cuprizone (CPZ) diet intoxication has also been widely used to evaluate the efficacy of remyelination-promoting treatments (Matsushima and Morell, 2001; Yamazaki et al., 2018). In the CPZ-induced model, clemastine enhanced remyelination in the corpus callosum, cerebral cortex, and hippocampus (Li et al., 2015). Overall, clemastine has been reported to have therapeutic effects on multiple white matter regions, including the corpus callosum, spinal cord, and internal capsule. Another relevant mouse model is the experimental autoimmune encephalomyelitis (EAE) model, in which demyelination is mediated by the immune system (Constantinescu et al., 2011; Robinson et al., 2014). Clemastine administration in the EAE model improved clinical scores and enhanced remyelination (Motawi et al., 2023; Ibrahim et al., 2024).

Different types of injury models have also been used to evaluate the therapeutic potential of clemastine. Spinal cord injury is a neurological disorder associated with traumatic damage to white matter tracts. In a rat model of spinal cord injury, clemastine was shown to improve functional recovery (Du et al., 2022; Tong et al., 2022). Traumatic brain injury is also known to affect white matter and cognitive function (Huntemer-Silveira et al., 2020; Strogulski et al., 2023). Clemastine was shown to enhance myelination of the cortex and hippocampus and improve cognitive function in a rat model of mild traumatic brain injury (Huang et al., 2024).

Stroke is a common cause of hypoxic brain injury (Wang et al., 2016; Youssef et al., 2021). Hypoxic conditions are known to induce oligodendrocyte loss in white matter regions (Dewar et al., 2003; Huang et al., 2023); promoting remyelination is therefore a potential therapeutic approach for resolving white matter damage associated with cerebral ischemia (Wang et al., 2016; Youssef et al., 2021; Huang et al., 2023; Fernandes et al., 2025). The middle cerebral artery occlusion (MCAO) model is one of the most widely used in stroke research (Ma et al., 2020; Li et al., 2023). Recently, it was reported that clemastine treatment preserved white matter integrity, promoted neuronal survival, and accelerated functional recovery after transient MCAO (Cheng et al., 2024). In models of neonatal and adult hypoxic brain injury, enhancing myelination with clemastine treatment led to functional recovery and improved motor coordination (Cree et al., 2018; Wang et al., 2018; Chen et al., 2021).

White matter changes have also been reported in patients with depressive disorders, bringing attention to the potential role of oligodendrocytes in depression and the stress response (Wang et al., 2014; van Velzen et al., 2020; He et al., 2022). Social isolation of adult mice impaired myelination in the prefrontal cortex (PFC) (Liu et al., 2012), while clemastine rescued the behavioral changes (Liu et al., 2016). The depressive-like behavior induced by social defeat stress in adolescent mice was also ameliorated by clemastine treatment (Shimizu et al., 2020). Chemotherapy is also reported to induce cognitive impairments, associated with the alteration of white matter integrity; clemastine was able to rescue such chemotherapy-induced abnormalities (Chen et al., 2022). Interestingly, clemastine administration in a mouse model of glaucoma was reported to attenuate optic nerve and retinal neuropathy by promoting remyelination by enhancing the differentiation of oligodendrocyte precursor cells (OPCs) (Liu et al., 2024). This suggests that promoting remyelination also has therapeutic potential for glaucoma. Finally, clemastine was reported to improve electrophysiological changes and promote peripheral myelin repair in a murine model of compression neuropathy (Lee et al., 2021). Overall, therapeutic effects of clemastine have been reported in various pathological animal models in preclinical studies (Table 1).

**Table 1:** Comparison of dose and route of clemastine administration in different animal models.

Model	Dose	Route	References
Lysolecithin-induced spinal cord demyelination (mouse)	10 mg/kg/day for 7 days or 14 days	Oral administration	Mei et al. (2014)
	10 mg/kg/day for 14 days	Gastric gavage	Jensen et al. (2018)
Lysolecithin-induced internal capsule demyelination (mouse)	10 mg/kg/day from 3 to 9 days post lesion (dpl) or 3–12 dpl	Intraperitoneal injection	Yamazaki et al. (2023)
Cuprizone model (mouse)	10 mg/kg/day for 3 weeks	Oral administration	Li et al. (2015)
EAE model (mouse)	10 mg/kg/day for 32 days	Oral gavage	Mei et al. (2016)
EAE model (rat)	5 mg/kg/day for 15 days	Oral administration	Motawi et al. (2023) Ibrahim et al. (2024)
Spinal cord injury model (rat)	10 mg/kg/day for 28 days	Oral gavage	Du et al. (2022)
	10 mg/kg/day for 7 or 14 days consecutively		Tong et al. (2022)
Traumatic brain injury model (rat)	10 mg/kg/day for 14 days	Oral gavage	Huang et al. (2024)
Middle cerebral artery occlusion (MCAO) model (rat)	5 mg/kg/day for 8 weeks	Oral administration	Cheng et al. (2024)
Neonatal hypoxic brain injury (mouse)	10 mg/kg/day for 7 days	Oral gavage	Cree et al. (2018)
	10 mg/kg/day from P3–P10 or P11–P18 for 8 days		Wang et al. (2018)
Adult hypoxic brain injury (mouse)	10 mg/kg/day for 4 weeks	Oral administration	Chen et al. (2021)
Social isolation model (mouse)	10 mg/kg/day for the last 2 weeks of social isolation	Gastric gavage	Liu et al. (2016)
Social defeat stress model (mouse)	10 mg/kg/day for the last 5 consecutive days of social defeat stress	Oral gavage	Shimizu et al. (2020)
Glaucoma (mouse)	10 mg/kg/day from the 7th day to 14th or 28th day after establishing glaucoma	Oral gavage	Liu et al. (2024)
Chemotherapy-induced white matter damage (mouse)	10 mg/kg/day for 2 weeks	Oral administration	Chen et al. (2022)
Compression neuropathy model (mouse)	10 mg/kg/day for 6 weeks	Intraperitoneal injection	Lee et al. (2021)

### 2.2 The potential molecular mechanisms underlying the therapeutic effects of clemastine

The M1 muscarinic acetylcholine receptor (CHRM1) was identified as the major target of clemastine by OPC culture studies; CHRM1 knockout mice exhibited accelerated remyelination and reduced axonal loss after EAE induction (Mei et al., 2016). In neonatal and adult hypoxic brain injury models, enhancing myelination with clemastine treatment led to functional recovery via CHRM1-mediated effects on OPCs (Cree et al., 2018; Wang et al., 2018). Therefore, CHRM1 is an important target receptor for clemastine administration. Recent reports have shown that clemastine administration in EAE mice activates F3/Contactin-1 through non-canonical Notch-1 signaling, while inhibiting p38 mitogen-activated protein kinase (MAPK)/NOD-like receptor protein-3 (NLRP3) signaling (Motawi et al., 2023; Ibrahim et al., 2024). Evidence has also shown that clemastine-induced activation of the glutathione S-transferase 4α (Gsta4)/4-hydroxynonenal (4-HNE) pathway promotes remyelination by oligodendrocytes (Carlström et al., 2020). Overexpression of Gsta4 has also been reported to contribute to the amelioration of the EAE phenotype (Carlström et al., 2020). Clemastine was shown to promote the differentiation of OPCs by activating extracellular signal-regulated kinase (ERK) signaling through the muscarinic receptor in the spinal cord injury model (Du et al., 2022; Tong et al., 2022). It has also been reported to reduce inflammation and induce the downregulation of NLRP3 and IL-1β, through the inhibition of the P38 signaling pathway in microglia (Xie et al., 2020). Clemastine enhanced the activity of H3K9 histone methyltransferases in PFC oligodendrocytes in a social isolation mouse model (Liu et al., 2016). Recently, Kamen et al. (2024) performed whole-cell patch-clamping and reported that clemastine induces an *N*-methyl-d-aspartate (NMDA) receptor-rich state in OPCs by altering membrane properties. Taken together, the molecular mechanisms of clemastine action in OPCs are currently being elucidated (Figure 1B).

## 3 Clinical studies, remaining questions, and future directions

Clinical trials of clemastine began in multiple sclerosis patients in 2014 (Green et al., 2017). In a double-blind, randomized, placebo-controlled, crossover trial (ReBUILD) in patients with relapsing multiple sclerosis, clemastine delayed the decline in visual-evoked potentials (Green et al., 2017). MRI analysis from the ReBUILD trial showed that normal-appearing regions of corpus callosum white matter exhibited increased derived myelin water fraction values after clemastine administration (Caverzasi et al., 2023). Because clemastine is safe to use, with a relatively low incidence of side effects, the results suggest promising benefits for the treatment of multiple sclerosis patients.

However, several questions remain in terms of human application. Clemastine treatment impaired myelination in the developmental stages in mice, despite an increase in the number of oligodendrocytes (Palma et al., 2022). A recent preprint clinical report also suggests that clemastine enhances pyroptosis and accelerates the advance of disability in progressive MS (Kocot et al., 2024). In addition, in a rabbit model of LPC-induced demyelination, short-term administration of clemastine reduced the number of OPCs, while delayed administration resulted in the accumulation of OPCs expressing markers of senescence (Cooper et al., 2024). However, long-term treatment increased the density of oligodendrocytes at the lesion site (Cooper et al., 2024). These results may reflect differences in oligodendrocyte numbers and the CNS environment between biological species. Optimization of the timing and duration of clemastine administration is therefore critical for achieving maximal therapeutic effects for different diseases. In addition, while clemastine may be more clinically effective when used in combination with existing treatments, such benefits are still unconfirmed. However, other muscarinic antagonists such as quetiapine and benztropine have also been identified as potential treatments in high-throughput screens (Deshmukh et al., 2013; Mei et al., 2014). Benztropine is currently used for Parkinson’s disease, while quetiapine is used as an atypical antipsychotic; both have been reported to promote OPC differentiation and myelin repair (Xiao et al., 2008; Zhang et al., 2012; Deshmukh et al., 2013; Wang et al., 2021). Clinical trials in multiple sclerosis patients have not yet been initiated for benztropine, but results from those of quetiapine have been reported. A phase I/II dose-finding study (NCT02087631) in patients with relapsing–remitting and progressive multiple sclerosis reported adverse effects including sedation and paraparesis (Metz et al., 2020). These results highlight the need to demonstrate the efficacy of low-dose quetiapine in preclinical studies. Recently, the selective muscarinic M1 receptor antagonists PIPE-359 and PIPE-307 were reported to improve clinical scores in an EAE model (Schrader et al., 2021; Poon et al., 2024; Chen et al., 2025). These novel drug candidates may attenuate side effects through the selective blockade of the M1 receptor. PIPE-307 has completed Phase I trials in healthy volunteers (NCT04725175 and NCT04941781) and is currently in Phase II in MS patients (NCT06083753) (Poon et al., 2024; Chen et al., 2025).

Recently, ApTOLL—a single-stranded DNA aptamer—and alpha-keto acids generated by the amino acid oxidase interleukin-4 induced 1 (IL4I1) have been reported as new drug candidates for MS (Fernández-Gómez et al., 2024; Hu et al., 2024). Previously, creatine, a nitrogenous organic acid, has also been reported to promote remyelination (Chamberlain et al., 2017; Rosko et al., 2023). These drug candidates are being evaluated in several types of demyelinating mouse models and may have different potential molecular mechanisms. However, these drugs have not been directly compared in their efficacy in promoting remyelination. Therefore, it may be important to compare their relative strengths against the expected effects of clemastine.

Meanwhile, in hypoxic–ischemic rats, clemastine was shown to promote exercise-induced motor improvement (Goto et al., 2025). Interestingly, synthetic MRI measurements of acute stroke patients suggested that higher myelin content in the brain leads to better prognosis (Toko et al., 2025). Therefore, the combination of clemastine administration and exercise may have a synergistic effect on rehabilitation. In the near future, clinical trials may be conducted combining rehabilitation and clemastine administration in stroke patients.

## 4 Conclusion

Over the past decade, the efficacy of clemastine for treating neurological disorders has been reported in various preclinical models, and the possibility of repurposing clemastine has been discussed. In this review, we have summarized previous reports on clemastine administration in models of white matter injury and neurological disease, and discussed the therapeutic effects and potential mechanisms of action. Taken together, clemastine has the potential to be repurposed for various neurological disorders. However, further investigation is needed to overcome outstanding questions, such as the optimization of the timing and duration of administration.
